# The Will Rogers phenomenon, breast cancer and race

**DOI:** 10.1186/s12885-021-08125-8

**Published:** 2021-05-17

**Authors:** Mary R. Nittala, Eswar K. Mundra, S. Packianathan, Divyang Mehta, Maria L. Smith, William C. Woods, Shawn McKinney, Barbara S. Craft, Srinivasan Vijayakumar

**Affiliations:** 1 Department of Radiation Oncology, University of Mississippi Medical Center, 350 West Woodrow Wilson, Jackson, MS 39213 USA; 2 Department of Surgery, University of Mississippi Medical Center, Jackson, MS USA; 3 Department of Medicine, University of Mississippi Medical Center, Jackson, MS USA

**Keywords:** Breast cancer, Racial disparities, Will Rogers phenomenon

## Abstract

**Background:**

The Will Rogers phenomenon [WRP] describes an apparent improvement in outcome for patients’ group due to tumor grade reclassification. Staging of cancers is important to select appropriate treatment and to estimate prognosis. The WRP has been described as one of the most important biases limiting the use of historical cohorts when comparing survival or treatment. The main purpose of this study is to assess whether the WRP exists with the move from the AJCC 7th to AJCC 8th edition in breast cancer [BC] staging, and if racial differences are manifested in the expression of the WRP.

**Methods:**

This is a retrospective analysis of 300 BC women (2007–2017) at an academic medical center. Overall survival [OS] and disease-free survival [DFS] was estimated by Kaplan-Meier analysis. Bi and multi-variate Cox regression analyses was used to identify racial factors associated with outcomes.

**Results:**

Our patient cohort included 30.3% Caucasians [Whites] and 69.7% African-Americans [Blacks]. Stages I, II, III, and IV were 46.2, 26.3, 23.1, and 4.4% of Whites; 28.7, 43.1, 24.4, and 3.8% of Blacks respectively, in anatomic staging (*p* = 0.043). In prognostic staging, 52.8, 18.7, 23, and 5.5% were Whites while 35, 17.2, 43.5, and 4.3% were Blacks, respectively (*p* = 0.011).

A total of Whites (45.05% vs. 47.85%) Blacks, upstaged. Whites (16.49% vs. 14.35%) Blacks, downstaged. The remaining, 38.46 and 37.79% patients had their stages unchanged.

With a median follow-up of 54 months, the Black patients showed better stage-by-stage 5-year OS rates using 8th edition compared to the 7th edition (*p* = 0.000). Among the Whites, those who were stage IIIA in the 7th but became stage IB in the 8th had a better prognosis than stages IIA and IIB in the 8th (*p* = 0.000). The 8th showed complex results (*p* = 0.176) compared to DFS estimated using the 7th edition (*p* = 0.004).

**Conclusion:**

The WRP exists with significant variability in the move from the AJCC 7th to the 8th edition in BC staging (both White and Black patients). We suggest that caution needs to be exercised when results are compared across staging systems to account for the WRP in the interpretation of the data.

## Background

Breast cancer is the most frequently diagnosed cancer in the world and the second leading cause of death in females [1]. African- American women have an increased rate of early disease onset and a higher mortality rate than any other racial group [2, 3]. Socioeconomic factor plays a major role in this disease disparity along with tumor biology or genetics [3, 4]. Cancer staging is an important tool to predict disease progression and for individual treatment design [5]. In 1959, the American Joint Committee on Cancer (AJCC), developed the TNM staging system; and, eight editions of the AJCC Cancer staging have been published since then [6, 7].

Breast cancer has been staged using AJCC TNM staging based on anatomic factors like the extent of the primary tumor (T), the status of adjacent nodes (N), and the presence of distant metastasis (M) [8]. However some reports have suggested that the 7th edition staging do not stratify patients accurately about the prognosis [9–12].

The Will Rogers phenomenon is an epidemiological paradox named after the remark made by the humorist Will Rogers about migration during the American economic depression of the 1930’s. Later in 1935, Alvan Feinstein proposed the name “Will Rogers phenomenon” to describe the effect of the “stage migration” in cancer patients where the stage-specific survival improved compared to an earlier cohort, even though the outcome of individual patients has not changed [13]. In oncology, Will Rogers phenomenon is often recognized as one of the most important biases limiting the use of historical control groups in experimental treatment trials and is an important issue to consider when interpreting the clinical studies results.

Over the past decades, the scientific developments led to a better understanding of breast cancer according to the expression of estrogen receptor (ER), progesterone receptor (PR), human epidermal growth factor receptor 2 (HER2) and proliferation marker (Ki-67) [6, 14, 15]. The close relationship of these receptors with the disease prognosis, treatment selection, and response to the treatment pose a challenge to the 7th AJCC cancer staging criteria for breast cancer. The American Society of Clinical Oncology (ASCO) [16], European Group on Tumor Markers (EGTM) [17], and the National Comprehensive Cancer Network (NCCN) [18] recommend the determination of ER, PR, and HER2 status for prognosis and treatment planning in all breast cancer patients. In addition to the traditional anatomic factors, the new 8th edition of the AJCC TNM classification for breast cancer incorporates grade, ER, PR, HER2, and multigene testing [19].

Controversy persists regarding how accurately the current 8th edition staging system can predict the breast cancer prognosis. The ability to evaluate the high-risk patients and cancer-related mortality is of great importance to treatment and follow up decisions [12, 20]. The primary objective of this retrospective study was to validate the prognostic value of the AJCC 8th edition staging system compared to the previous AJCC 7th staging system and its association with race and overall survival (OS) using a single institute breast cancer research database.

The Will Rogers phenomenon / stage migration has been shown not only in breast cancer but also in lung, prostate and other cancers [13, 21–24]. Stage migration difference between Whites and Blacks has been well demonstrated in prostate cancer but not demonstrated in other cancers. In prostate cancer, because of the availability of good biomarkers like prostate- specific antigen “PSA”, it has been easy to demonstrate Will Rogers phenomenon. Since, prostate cancer prevalence is higher in blacks, this phenomenon has been studied extensively [25, 26]. However, in breast cancer to the best of our knowledge, no study has addressed the Will Rogers phenomenon differences between Whites and Blacks.

In this study, we not only demonstrate the Will Rogers phenomenon among total population in breast cancer but also show differences in the phenomenon among Whites and Blacks.

We hypothesize that because of the differences between AJCC – 7 and AJCC – 8, stage migration is likely to have occurred. In addition we hypothesize that the extent of such stage migration is likely to be different for Whites and Blacks. Because of our institution’s unique geographical location and being a safety-net academic institution, our populations spread is likely to study this subject with statistical validity to draw conclusions.

## Methods

### Study design and participants

**Fig. 1:**
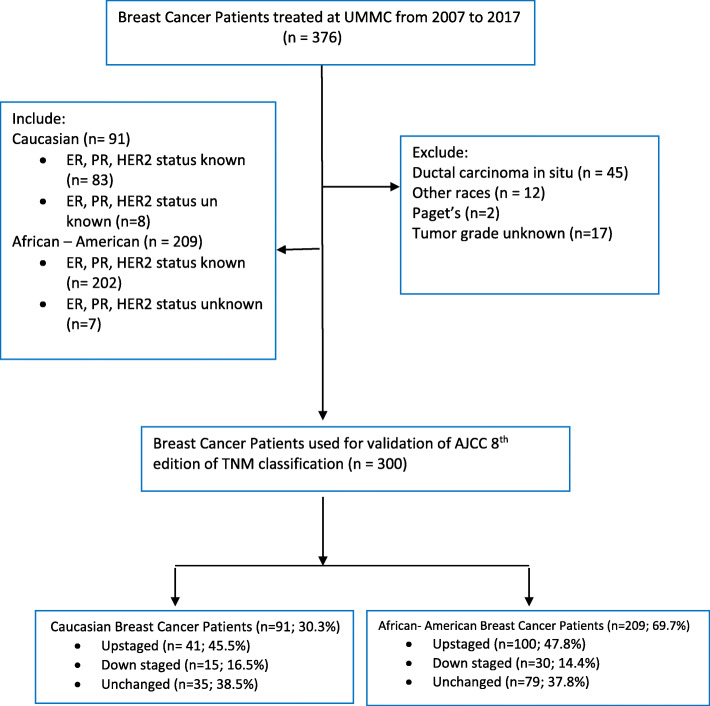
Flow chart for Breast Cancer Patients Cohort selection. UMMC, University of Mississippi Medical Center; ER, estrogen receptor; PR, progesterone receptor, HER2, human epidermal growth factor receptor 2; n, number; %, percentage

This retrospective study includes a total of 376 breast cancer women diagnosed and treated between 2007 and 2017 at the University of Mississippi Medical Center (UMMC), Jackson, Mississippi, USA. Institutional review board approval was obtained and a browser-based database tool, research electronic data capture (RedCap) was used to gather and store the patient’s information in password-protected computers. Seventy-six patients with unknown pathology staging, other races (other than Caucasians [Whites] or African Americans [Blacks]) and ductal carcinoma in situ were excluded from the study. A total of 300 breast cancer women were included in this analysis (shown in Fig. 1).

### Data collection

Epidemiological, clinical, demographic, treatment and outcome data was obtained from RedCap. The patients had all been stratified by race according to the 7th AJCC anatomic staging system. The following patient characteristics were collected: date of diagnosis, age, race, body mass index (BMI), tumor grade, receptors (ER, PR, HER2) status and survival months. Patients were then restaged using the 8th AJCC prognostic staging system to estimate the effect of stage migration (upstaged, downstaged/ unchanged) on the stage specific survival compared to the earlier cohort. All data was collected, checked, analyzed, and interpreted by the postdoc research fellow (MN).

### Definitions

The Will Rogers phenomenon refers to the “improved” survival of patients with cancer or other diseases by either reclassifying them into different prognostic groups, recognizing subtle disease manifestations, or by using diagnostic modalities that allow the disease to be diagnosed at an earlier stage. Disease-free survival (DFS) is defined as the number that predicts the chances of staying free of disease or cancer after a particular treatment. Overall survival (OS) is defined by the number of days from the date of initial diagnosis until the date of death/ the last contact. The censored cases defined as the patients without death at the time of the last follow up.

### Statistical analysis

The Pearson’s chi-square test was used to discover the relationship between the two categorical variables as well as the stage migration changes between the two races. The respective *p*-values were recorded. Kaplan-Meier method was used to estimate the OS rates and the univariate significance of differences among survival curves calculated by the Mantel-Cox log-rank test. The five-year OS rates by AJCC stage group and race were estimated from the cumulative proportion surviving at the particular time (survival table). *P* values ≤0.05 were considered significant. To indicate Will Rogers phenomenon, the improved survival by stage in AJCC 8th edition vs. AJCC 7th edition is recorded. The co-variables associated with the OS and DFS were determined by the bi and multivariate Cox regression model. The Black race is considered the referent group for the analyses. Hazards ratio (HR) was used to estimate time to event outcome with associated 95% confidence intervals (CIs) and *P* values ≤0.05 were considered statistically significant. Data was analyzed using SPSS 24.0 software (IBM, Armonk, NY, USA).

## Results

### Patient characteristics

A total of 300 primary breast cancer patients treated at UMMC between 2007 and 2017 were identified. Out of 300 patients, 91 (30.3%) were Whites and 209 (69.7%) Blacks. The baseline characteristics of these patients (Table 1) had a median age of 62 years [y] (range, 34 to 92 y) and Black women were diagnosed with breast cancer at a young age compared to Whites (32.6% vs.19.8%; *p* = 0.025). Black breast cancer women presented were with higher BMI levels compared to Whites (60.3% vs. 41%; *p* = 0.007).

**Table 1:** Baseline characteristics of included breast cancer patients (*n* = 300)

	White (***n*** = 91) 30.3%	Black (***n*** = 209) 69.7%	All Patients (n = 300) 100%	***p***-Value
**Age**				
≤ 39	2 (2.2%)	6 (2.9%)	8 (2.7%)	0.074
40–59	16 (17.6%)	62 (29.7%)	78 (26.0%)	
60–79	51 (56.0%)	110 (52.6%)	161 (53.7%)	
≥ 80	22 (24.2%)	31 (14.8%)	53 (17.7%)	
**BMI**				
Normal	15 (16.5%)	29 (13.9%)	44 (14.7%)	0.058
Overweight	36 (39.6%)	54 (25.8%)	90 (30.0%)	
Obese	30 (33.0%)	90 (43.1%)	120 (40.0%)	
Morbidity	10 (11.0%)	36 (17.2%)	46 (15.3%)	
**Grade**				
I	29 (32.2%)	40 (19.1%)	69 (23.0%)	**0.035**
II	32 (35.6%)	75 (35.9%)	107 (35.7%)	
III- IV	30 (33.0%)	94 (45.0%)	124 (41.3%)	
**ER status**				
Positive	68 (74.7%)	137 (65.6%)	205 (68.3%)	**0.004**
Negative	20 (22.0%)	72 (34.4%)	92 (30.7%)	
Unknown	3 (3.3%)	0 (0.0%)	3 (1.0%)	
**PR status**				
Positive	59 (64.8%)	101 (48.3%)	160 (53.3%)	**0.015**
Negative	30 (33.0%)	106 (50.7%)	136 (45.3%)	
Unknown	2 (2.2%)	2 (1.0%)	4 (1.3%)	
**HER2 status**				
Positive	26 (28.6%)	35 (16.7%)	61 (20.3%)	0.053
Negative	62 (68.1%)	169 (80.9%)	231 (77.0%)	
Unknown	3 (3.3%)	5 (2.4%)	8 (2.7%)	
**7th AJCC TNM stage**				
Stage IA	42 (46.2%)	60 (28.7%)	102 (34.0%)	**0.043**
Stage IIA	15 (16.5%)	54 (25.8%)	69 (23.0%)	
Stage IIB	9 (9.9%)	36 (17.2%)	45 (15.0%)	
Stage IIIA	7 (7.7%)	20 (9.6%)	27 (9.0%)	
Stage IIIB	11 (12.1%)	17 (8.1%)	28 (9.3%)	
Stage IIIC	3 (3.3%)	14 (6.7%)	17 (5.7%)	
Stage IV	4 (4.4%)	8 (3.8%)	12 (4.0%)	
**8th AJCC TNM stage**				
Stage IA	25 (27.5%)	38 (18.2%)	63 (21.0%)	**0.011**
Stage IB	23 (25.3%)	35 (16.7%)	58 (19.3%)	
Stage IIA	8 (8.8%)	24 (11.5%)	32 (10.7%)	
Stage IIB	9 (9.9%)	12 (5.7%)	21 (7.0%)	
Stage IIIA	4 (4.4%)	30 (14.4%)	34 (11.3%)	
Stage IIIB	3 (3.3%)	26 (12.4%)	29 (9.7%)	
Stage IIIC	14 (15.4%)	35 (16.7%)	49 (16.3%)	
Stage IV	5 (5.5%)	9 (4.3%)	14 (4.7%)	

*White* Caucasians, *Black* African American, *BMI* body mass index, *ER* estrogen receptor, *PR* progesterone receptor, *HER2* human epidermal growth factor receptor 2, *AJCC* American Joint Committee on Cancer, *n* Number

When the receptor characteristics of this study population were measured, the most common subtype was ER+/PR+/HER2-, accounting 33.3% of Whites and 66.7% of Blacks, respectively (*p* = 0.336). The Blacks were significantly less likely to have positive tumor receptors of ER (65.6% vs. 74.7%) and PR (48.3% vs. 64.8%) compared to White women. Stages I, II, III, and IV accounted for 46.2, 26.3, 23.1, and 4.4% of Whites and 28.7, 43.1, 24.4, and 3.8% of Blacks respectively, in 7th AJCC anatomic staging (*p* = 0.043). In 8th AJCC prognostic staging, 52.8, 18.7, 23, and 5.5% were Whites while 35, 17.2, 43.5, and 4.3% were Blacks, respectively (*p* = 0.011). Black women were significantly more likely to be diagnosed at a later stage, notably at stage IIB and higher in 7th AJCC anatomic staging and notably at stage IIIA and higher in 8th AJCC prognostic staging.

A total number of 41 Whites (45.1%) upstaged compared to 100 Black (47.9%) patients. Fifteen White patients (16.5%) and 30 Black patients (14.4%) were downstaged. Of the remainder, 35 White (38.5%) and 79 Black (37.8%) patients had their stages unchanged (*P* = 0.859) (Table 2). The greatest changes in stage for 8th AJCC were in stage IB (rise by 19.3%), IA (drop by 13%), IIA (drop by 12.3%), and IIB (drop by 8%) compared to the 7th AJCC anatomic staging. The stage IB changes resulted from two components: the stage IIA (12.3%) and IIB (8%), downstaged to IB; and stage IA (13%) upstaged to IB from the 7th AJCC anatomic staging system to the 8th AJCC prognostic staging system.

**Table 2:** Descriptive statistics of upstaging, down staging and unchanged staging

	White (***n*** = 91) 30.3%	Black (***n*** = 209) 69.7%	All patients (***n*** = 300) 100%	***p***-Value
Upstaged	41 (45.0%)	100 (47.8%)	141 (47.0%)	0.859
Down staged	15 (16.5%)	30 (14.4%)	45 (15.0%)	
No Change	35 (38.5%)	79 (37.8%)	114 (38.0%)	

*AJCC* American Joint Committee on Cancer, *Ed* Edition

### Kaplan-Meier curves for overall survival and disease-free survival

**Fig. 2:**
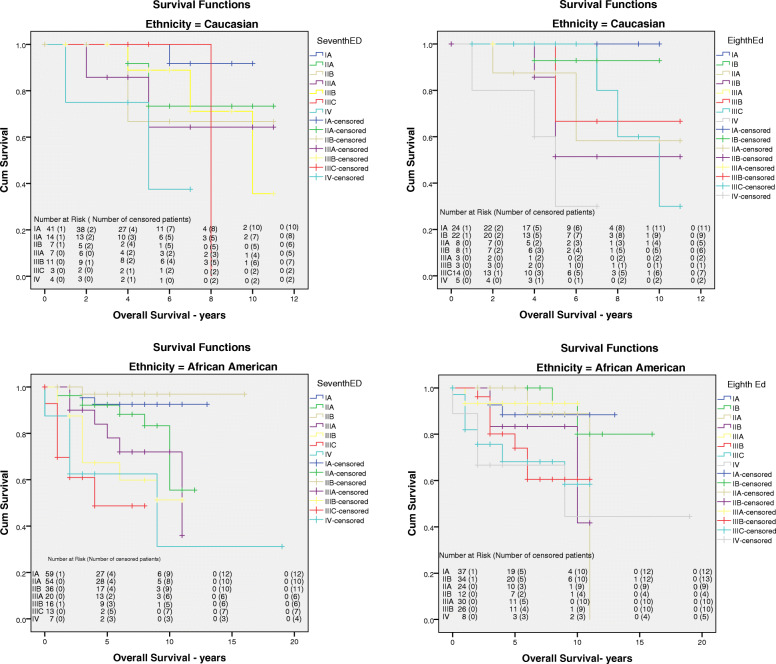
The Kaplan- Meier survival curves (years) for AJCC 7th anatomic staging and AJCC 8th prognostic staging for Caucasian and African- American women diagnosed with breast cancer. AJCC, American Joint Committee

The Kaplan-Meier survival curves for the length of time after initial diagnosis until occurrence of the primary endpoint (shown in Fig. 2). There was a significant difference in survival times among both races between the 7th AJCC anatomic staging system and 8th AJCC prognostic staging system (*p* = 0.000). Univariate analysis by Kaplan-Meier demonstrated that White patients with stage IIIA had a worse prognosis than those with stage IIIB within the 7th AJCC anatomic staging system (64.3% vs.85.9%; p = 0.000) at a median follow-up of 58 months (range 4 to 235 months) (shown in Fig. 2). The number of patients at risk and of censored patients for each stage between both races in different staging system were depicted in Kaplan-Meier curves (shown in Fig. 2).

The five-year OS rates by stage (Table 2) indicate that the Black patients showed better stage-by-stage rates using 8th edition compared to the 7th edition, suggesting a manifestation of the WRP. Among the White patients, those who were stage IIIA in the 7th edition but became stage IB in the 8th had a better prognosis than stages IIA and IIB in the 8th edition (*p* = 0.000). For Black patients, stage IIIA, IIIB, IIIC, and IV (93.3, 74, 68.1, and 66.7%) all demonstrated better prognoses in the 8th edition when compared to the 7th edition (*p* = 0.000).

**Fig. 3:**
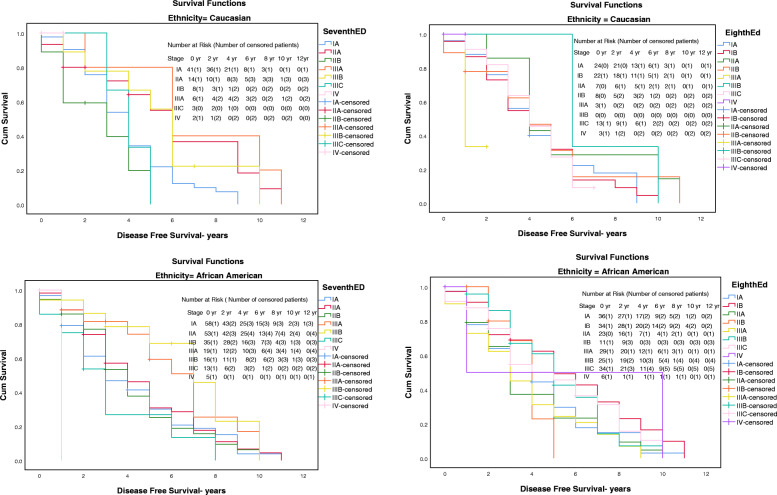
The Kaplan- Meier disease-free survival curves (years) for AJCC 7th anatomic staging and AJCC 8th prognostic staging for Caucasian and African- American women diagnosed with breast cancer. AJCC, American Joint Committee

In terms of DFS, the 8th edition’s clinical staging showed complex results (*p* = 0.176) compared to DFS estimated using the 7th^’^s anatomic staging system (*p* = 0.004) (Table 2). For White patients, stages IA, IB, IIB, and IIIC (31.1, 31.9, 31.1, and 27.3%) all recorded better DFS when using the 8th edition while for Black patients, only those with stages IB and IIIC (49.2 and 45.5%) showed better DFS in the 8th edition compared to the 7th (shown in Fig. 3) (Table 3).

**Table 3:** Five-year overall survival & five-year disease free survival of the breast cancer study population

	5-year overall survival (***p*** = 0.000)				5-year disease free survival (***p*** = 0.000)			
	Caucasian		African American		Caucasian		African American	
	AJCC-7th Ed	AJCC -8th Ed	AJCC-7th Ed	AJCC -8th Ed	AJCC-7th Ed	AJCC -8th Ed	AJCC-7th Ed	AJCC - 8th Ed
Stage IA	91.7%	100.0%	92.5%	88.4%	22.0%	31.1%	30.1%	29.7%
Stage IB	..	92.9%	..	100.0%	..	31.9%	..	49.2%
Stage IIA	73.3%	58.3%	88.2%	88.9%	54.9%	28.6%	30.7%	23.3%
Stage IIB	66.7%	51.4%	96.9%	83.3%	0.00%	31.1%	25.1%	0.00%
Stage IIIA	64.3%	..	78.0%	93.3%	40.0%	_	59.2%	24.2%
Stage IIIB	85.9%	66.7%	59.8%	74.0%	55.6%	33.3%	68.6%	42.6%
Stage IIIC	0.00%	80.0%	48.8%	68.1%	0.00%	27.3%	13.4%	45.5%
Stage IV	37.5%	30.0%	31.3%	66.7%	0.00%	0.00%	0.00%	0.00%

### Cox regression analysis for overall survival

The hazard ratios [HR] comparing racial differences (Blacks vs. Whites) about the risk of death was calculated by bivariate Cox regression (Table 4). The Black breast cancer patients with overweight BMI, showed double the risk of mortality (HR 2.83, 95% CI, 1.01–7.91; *p* = 0.046) compared to White patients.

**Table 4:** Bivariate cox regression analysis

African American vs. Caucasian		
	HR (95% CI)	***P*** Value
**Age**	0.90 (0.60–1.34)	0.623
**Grade**	1.38 (0.95–2.02)	0.087
**ER status**	1.52 (0.90–2.58)	0.115
**PR status**	1.26 (0.75–2.12)	0.373
**HER 2 status**	1.74 (0.88–3.42)	0.109
**BMI (kg/ m**^**2**^**)**		
Normal (18.5–24.9)	1	
Overweight (25–29.9)	2.83 (1.01–7.91)	**0.046**
Obese (30–39.9)	1.12 (0.39–3.20)	0.831
Morbidity (> 40)	1.18 (0.43–3.20)	0.746
**7th AJCC TNM stage**		
Stage IA	1	
Stage IIA	0.23 (0.09–0.58)	**0.002**
Stage IIB	0.76 (0.41–1.38)	0.375
Stage IIIA	0.23 (0.07–0.80)	**0.020**
Stage IIIB	1.30 (0.66–2.55)	0.444
Stage IIIC	1.64 (0.87–3.08)	0.119
Stage IV	3.38 (1.64–6.94)	**0.001**
**8th AJCC TNM stage**		
Stage IA	1	
Stage IB	0.34 (0.12–0.96)	**0.042**
Stage IIA	0.32 (0.11–0.91)	**0.033**
Stage IIB	0.83 (0.33–2.07)	0.680
Stage IIIA	1.81 (0.83–3.94)	0.130
Stage IIIB	0.45 (0.13–1.60)	0.222
Stage IIIC	1.84 (0.95–3.59)	0.070
Stage IV	1.96 (1.11–3.47)	**0.020**

*BMI* body mass index, *ER* estrogen receptor, *PR* progesterone receptor, *HER2* human epidermal growth factor receptor 2, *AJCC* American Joint Committee on Cancer, *HR* hazard ratio, *CI* confident interval

The risk estimates for 7th AJCC staging indicate that Black breast cancer patients showed significant decreased risk for stage IIA (HR 0.23, 95% CI, 0.09–0.58; *p* = 0.002), IIB (HR 0.76, 95% CI, 0.41–1.38; *p* = 0.375), and IIIA (HR 0.23, 95% CI, 0.07–0.80; *p* = 0.020). Followed by increased risk of death for stages IIIB (HR 1.30, 95% CI, 0.66–2.55; *p* = 0.444), IIIC (HR 1.64, 95% CI, 0.87–3.08; *p* = 0.119), and three times significantly higher risk of death for stage IV (HR 3.38, 95% CI, 1.64–6.94; *p* = 0.001), compared to the White breast cancer patients.

The risk estimates for 8th AJCC staging indicate that Black breast cancer patients showed significant decreased risk for stage IB (HR 0.34, 95% CI, 0.12–0.96; *p* = 0.042), IIA (HR 0.32, 95% CI, 0.11–0.91; *p* = 0.033); decreased risk for stage IIB (HR 0.83, 95% CI, 0.33–2.07; *p* = 0.680) and stage IIIB (HR 0.45, 95% CI, 0.13–1.60; *p* = 0.222). Showed increased risk of death for stages IIIA (HR 1.81, 95% CI, 0.83–3.94; *p* = 0.130), stage IIIC (HR 1.84, 95%CI, 0.95–3.59; *p* = 0.070), and twice significantly higher risk of death for stage IV (HR 1.96, 95% CI, 1.11–3.47; *p* = 0.020), compared to the White breast cancer patients.

The multivariate Cox regression was built in which all the significant variables, BMI, 7th AJCC staging and 8th AJCC staging were added in two separate models (Tables 5 and 6). Black breast cancer patients were twice the increased risk of death with higher BMI compared to White patients. The multivariate risk estimates for 7th AJCC staging indicate that Black breast cancer patients showed significant decreased risk for stage IIA (HR 0.26, 95% CI, 0.10–0.63; *p* = 0.003), IIIA (HR 0.24, 95% CI, 0.07–0.81; *p* = 0.022), and three times significantly higher risk of death for stage IV (HR 3.70, 95% CI, 1.79–7.65; *p* = 0.000), compared to the White breast cancer patients (Table 5).

**Table 5:** Multivariate cox regression analysis for 7th AJCC TNM stage

African American vs. Caucasian		
	HR (95% CI)	***P*** Value
**BMI (kg/ m**^**2**^**)**		
Normal (18.5–24.9)	1	
Overweight (25–29.9)	1.79 (0.60–5.23)	0.289
Obese (30–39.9)	0.92 (0.31–2.66)	0.878
Morbidity (> 40)	1.18 (0.43–3.23)	0.741
**7th AJCC TNM stage**		
Stage IA	1	
Stage IIA	0.26 (0.10–0.63)	**0.003**
Stage IIB	0.76 (0.42–1.40)	0.392
Stage IIIA	0.24 (0.07–0.81)	**0.022**
Stage IIIB	1.38 (0.69–2.76)	0.350
Stage IIIC	1.55 (0.82–2.93)	0.175
Stage IV	3.70 (1.79–7.65)	**0.000**

*BMI* body mass index, *AJCC* American Joint Committee on Cancer, *HR* hazard ratio, *CI* confident interval

**Table 6:** Multivariate cox regression analysis for 8th AJCC TNM stage

African American vs. Caucasian		
	HR (95% CI)	***P*** Value
**BMI (kg/ m**^**2**^**)**		
Normal (18.5–24.9)	1	
Overweight (25–29.9)	1.84 (0.62–5.45)	0.266
Obese (30–39.9)	1.02 (0.35–2.99)	0.964
Morbidity (> 40)	1.07 (0.38–2.99)	0.885
**8th AJCC TNM stage**		
Stage IA	1	
Stage IB	0.11 (0.29–0.48)	**0.003**
Stage IIA	0.11 (0.02–0.44)	**0.002**
Stage IIB	0.28 (0.07–1.05)	0.059
Stage IIIA	0.56 (0.18–1.77)	0.330
Stage IIIB	0.15 (0.03–0.77)	**0.023**
Stage IIIC	0.61 (0.21–1.76)	0.366
ableStage IV	0.66 (0.24–1.75)	0.403

*BMI* body mass index, *AJCC* American Joint Committee on Cancer, *HR* hazard ratio, *CI* confident interval

All the stages in the 8th AJCC staging group showed decreased risk for Black patients compared to Whites. Stage IB (HR 0.11, 95% CI, 0.29–0.48; p = 0.003), IIA (HR 0.11, 95% CI, 0.02–0.44; *p* = 0.002), and IIIB (HR 0.15, 95% CI, 0.03–0.77; *p* = 0.023) showed significantly decreased risk.

## Discussion

The Will Rogers phenomenon refers to the “improved” survival of patients with cancer or other diseases by either reclassifying them into different prognostic groups, recognizing subtle disease manifestations, or by using diagnostic modalities that allow the disease to be diagnosed at an earlier stage [20]. In other words, the Will Rogers phenomenon describes an apparent improvement in outcome for a group of patients with no actual improvement for any individual patient but due to tumor stage or tumor grade reclassification. This phenomenon is based on a remark made by the humorist-philosopher Will Rogers “When the Okies left Oklahoma and moved to California, they raised the average intelligence level in both states” [13, 27].

Feinstein AR, et al. first used the term "Will Rogers phenomenon" to describe the effect of the “stage migration” observed in 1977 lung cancer patients cohort [13], the stage-specific survival improved in a 1997 cohort compared to an earlier cohort [20]. Feinstein AR, et al. suggested that the improvement of stage-specific OS rates among lung cancer patients is a statistical artifact, and can be resolved with proper intensive scientific attention [13]. The study by Chee KG, et al. also supported the notion that stage migration is responsible for the improvement of survival for patients with stage III and stage IV non-small cell lung cancer, demonstrating that the Will Rogers phenomenon is an important issue to consider when interpreting the clinical studies results [28].

Woodward WA and her colleagues reported that 5–10 survival rates for stage II breast cancer women according to 1988 guidelines were 72 and 53%, respectively. The same women showed 86 and 75% OS when classified according to the 2003 guidelines. They also reported that 31% of women with 1988- diagnosed stage IIa disease, 54% of stage IIb and 38% of stage IIIa, moved into higher stage groups, implying that comparisons of survival data between patients staged with different systems will be inaccurate and must include tumor characteristics that will result in significant changes in reported outcome by stage [29, 30]. Their study concluded that the revised AJCC staging guidelines raised significant improvements in stage-specific survival of breast cancer patients and careful attention be given to the Will Rogers phenomenon for accurate decision making for improved new treatment strategies [30].

Kim, et al. reported that breast cancer patient cohorts staged under AJCC5, showed poorer 10-year OS who were upstaged under AJCC6, (81 vs. 78% IIA, 73 vs. 65.4% IIB, 69.7 vs. 54.8% IIIA), demonstrating that upstaging reports more accurate disease outcomes and suggested to re-evaluate the nodal involvement impact upon new staging system [31]. It is very important to standardize the AJCC staging when comparing the stage at presentation and the stage-specific survival over a time period [20, 29, 31].

Albersten PC, et al. reported a decline in the low-grade prostate cancer incidence rate, which seems to be the result of Gleason score reclassification (average upgrade of 0.85 points; *p* < 0.001) over the past two decades improving the clinical outcomes (26% reduction in mortality; *p* = 0.012) reflecting the Will Rogers phenomenon [21].

The Will Rogers phenomenon is also reported by de Manzoni, et al. in melanoma and carcinoma of the stomach with stage migration increased (39.1%) with the level of nodal involvement (pN1 tier 3.8% vs. 50% in pN3 tier), and with depth of tumor invasion (pT1 class 1.5% vs. 15.7% in pT3/pT4 class) [32].

George S, et al. reported that stage migration along with biological predetermination is a significant factor in colorectal cancer patient’s survival outcomes [33]. Champion GA, et al. reported that improved diagnostic imaging will lead to improved stage-specific survival (stage I, III improved + 3%, IV improved + 11%) without any change in overall survival in laryngeal cancer patients [34].

We are the first to report a large cohort of Black population with the manifestation of the Will Rogers phenomenon, also the first to report the differences in the Will Rogers phenomenon between Blacks and Whites**.** Ours is one of the many studies documenting Will Rogers phenomenon between AJCC 7th anatomic staging system and AJCC 8th prognostic staging system among breast cancer patients. Shao N, et al. demonstrated the 8th AJCC prognostic accuracy to be superior to the anatomic 7th AJCC staging system in breast cancer patients using SEER database. They reported 3-year OS for stage IIIB (68.5% vs. 75.5%) to be inferior to IIIC in 7th AJCC staging system, but this inconsistency was not seen in the 8th AJCC system (85.5% vs. 69.9%). Thus concluding the higher the patient is staged, the poorer the prognosis will be seen in the 8th prognostic staging system [11]. Wong RX, et al. demonstrated similar findings confirming the prognostic accuracy of 8th AJCC over the 7th AJCC staging system in predicting outcomes among the Asian population. They reported the 8th AJCC prognostic staging system had better 5-year cancer-specific survival (0.79 vs. 0.77) compared to the 7th AJCC anatomic staging system [35].

Many studies show that Black women who were diagnosed with breast cancer have a significantly increased risk of death compared with White women [13–15]. Many factors contribute to race-related disparities in breast cancer outcomes [17, 18]; tumor biology, age, BMI, and AJCC tumor staging (7th & 8th) may further explain the significant correlation of the race and survival outcomes. Another factor to consider is the Will Rogers phenomenon. The current study reports that Black patients were diagnosed at a younger age with high-grade breast cancer than White patients (44.6 vs. 23.3%), and Black breast cancer patients at younger ages with high-grade showed worse survival compared to Whites. There were also differences in the Will Rogers phenomenon between the races.

The poorer prognosis for obese Black patients has been reported in a previous study [27]; our study confirms the double the risk of death for Black patients whose BMI is at an overweight level compared to White patients. Obesity is linked with later tumor stage at the time of diagnosis [20] and poorer survival [14, 27]; our study reports that 45% of Black and 33% of White patients were presented with high tumor grade.

Black women were less likely to have positive hormone receptors (ER/PR/HER2), more likely to have negative hormone receptors, and significant racial differences were noted in histologic categories [14], our study reports similar findings. Shao N et al. reported that the prognostic accuracy of the 8th AJCC staging system to be superior to the 7th AJCC [13], similar findings were reported by the Abdel- Rahman using the SEER research database [28]. Our retrospective study reports, when breast cancer patients restaged from 7th AJCC to 8th AJCC staging system, a total percent of Whites (45.1% vs. 47.9%) upstaged, (16.5% vs. 14.4%) downstaged and (38.5% vs. 37%) remained unchanged compared to Black patients with no significant difference between races.

In our study, 5-year OS rates by stage indicate that for White breast cancer patients, IIIC was still inferior to stage IV in the 7th AJCC staging system but this inconsistency was reproduced at IIIB and IV in the 8th AJCC prognostic staging system. Among the Black breast cancer patients, IIA was inferior to stage IIB in the 7th AJCC staging system with no inconsistency (IIA & IIB) but inconsistent (IIB & IIIA) in the 8th AJCC prognostic system. Stage IIIA among White patients in the 7th AJCC migrated to IB and have a worse prognosis than IIA or IIB in the 8th AJCC prognostic system. In contrast, IIIA patients among Black patients had better survival than IIA or IIB in the 8th AJCC prognostic system. A similar inconsistency was observed in the 5-year DFS between both races when restaged from 7th AJCC to 8th AJCC.

## Summary and conclusion

Our analyses suggest that the Will Rogers phenomenon exists in the move from the AJCC 7th to the 8th edition in breast cancer staging in both White and Black patients. However, there were significant variabilities between the races in the extent of their manifestation. We suggest that caution needs to be exercised when results are compared across staging systems in view of the Will Rogers Phenomenon in the interpretation of the data for both Caucasians and African Americans.

## Data Availability

This study does not contain any sequence/ expression data, protein/molecule characterizations, annotations, and taxonomy data to be deposited in a public repository. Patient identifiers were removed before extracting data and will be shared upon reasonable request to Mary R Nittala.

## Abbreviations

AJCC: American Joint Committee on Cancer
ASCO: The American Society of Clinical Oncology
BC: Breast Cancer
Black: African Americans
BMI: Body Mass Index
CI: Confident Interval
DFS: Disease- Free Survival
EGTM: European Group on Tumor Markers
ER: Estrogen Receptor
HER2: Human Epidermal Growth Factor Receptor 2
HR: Hazard Ratio
M: Distant Metastasis
N: Adjacent Nodes
NCCN: National Comprehensive Cancer Network
OS: Overall Survival
PR: Progesterone Receptor
RedCap: Research Electronic Data Capture
T: Primary Tumor
UMMC: University of Mississippi Medical Center
White: Caucasians
WRP: Will Rogers Phenomenon
